# Wide-neck renal artery aneurysm: parenchymal sparing endovascular treatment with a new device

**DOI:** 10.1186/1471-2490-14-42

**Published:** 2014-05-28

**Authors:** Michele Rossi, Gianluca Maria Varano, Gianluigi Orgera, Alberto Rebonato, Florindo Laurino, Cosimo De Nunzio

**Affiliations:** 1 Department of Radiology Interventional Radiology Unit, Ospedale Sant’Andrea, University “La Sapienza”, Via di Grottarossa 1035/1039 00189, Rome, Italy; 2 Department of Surgery, Radiology, and Odontostomatology Sciences, Santa Maria della Misericordia University Hospital, University of Perugia, Sant’Andrea delle Fratte, 06129 Perugia, Italy; 3 Division of Urology, Ospedale Sant’Andrea, University “La Sapienza”, Roma Via di, Grottarossa 1035/1039 00189, Rome, Italy

**Keywords:** Renal artery aneurysm, Visceral artery aneurysm, Wide-neck aneurysm, Embolization, Penumbra, Microcoil embolization

## Abstract

**Background:**

Renal artery aneurysm is a rare disorder with a high mortality rate in the event of rupture, the most frequent complication, which can also occur in lesions smaller than those indicated for treatment by current criteria. Surgery is still the first-line treatment, although a growing trend toward endovascular management of visceral artery aneurysms has emerged because of the high efficacy and low invasiveness that has been demonstrated by several authors. Treatment of wide-necked aneurysms and, depending on location, those at renal artery bifurcations or distal branches is more complex and may require invasive surgical techniques, such as bench surgery.

**Case presentation:**

We describe the successful use of a new neurointerventional coil to treat an enlarging wide-necked segmental-branch renal aneurysm in an elderly woman who was not a candidate for surgery because of several comorbidities.

**Conclusions:**

The technique described allowed safe, successful treatment of a wide-necked aneurysm in an unfavorable vascular territory, reducing the risk of downstream artery embolization and consequent parenchymal damage and decreased renal function. In similar cases, other endovascular devices have often proven to be ineffective at nephron sparing. To validate the safety and efficacy of this system, more cases treated in this manner should be studied.

## Background

Renal artery aneurysm (RAA) is a rare disorder, occurring in 0.01%–1.3% of the general population. It represents 1% of all aneurysms but is the second most common visceral artery aneurysm, accounting for approximately 25% [1,2].

RAAs are classified into four categories: saccular (70%); fusiform (22.5%); dissecting (7.5%); and mixed, including microaneurysms [3]. Saccular aneurysms, the most common, typically occur at the primary or secondary bifurcations of renal arteries [4]; more rarely they are located intraparenchymally in a segmental artery or more distal branches. Etiologies for saccular aneurysms include atherosclerosis, fibromuscular dysplasia, and neurofibromatosis. RAAs are often noted incidentally but can also be accompanied by hypertension, hematuria, or pain. Hypertension related to RAA remains a controversial topic; however, most case series on RAA treatment report improvement or resolution of hypertension after treatment [2].

The indication for treatment is currently based on a size threshold that continues to be debated, although most authors believe that aneurysms 2 cm or less in diameter do not require treatment. However, the decision to treat an aneurysm should be made not only on the basis of size, but also after considering other issues, such as the presence of clinical symptoms (hematuria, hypertension, back pain, and renal infarction), anatomical and morphological characteristics (location, wall calcification, enlarging lesion), and general clinical features (life expectancy, comorbidities, planned pregnancy). Finally, it should be considered that the mortality rate for spontaneous rupture is about 80% [5]. This outcome has also been described for aneurysms smaller than 1 cm [6,7].

Many treatment options are currently available. Surgical management still represents the standard of care, particularly for aneurysms located at the renal artery bifurcation as well as those involving distal branches [8]. Surgical repair may involve in situ or extracorporeal bench surgery [6], and may require a long operation time and have substantial perioperative complications such as unplanned nephrectomy, with a failure rate of 6.6%. After successful early surgical repair, there is a reintervention rate of 5.8% resulting from such complications as anastomotic stenosis or graft thrombosis [9].

Despite the relative success of surgical repair of symptomatic aneurysms, there is a growing trend toward endovascular alternatives over open procedures because of their low invasiveness and reduced morbidity. A recent review of endovascular treatment reported good clinical and angiographic success rates without major complications, loss of kidney function, or nephrectomy [10]. However, no large case series or case reports with long-term follow-up are currently available.

Several endovascular embolization techniques have recently been developed to manage aneurysms with heterogeneous anatomical features. Indeed, while narrow-necked saccular aneurysms can be successfully treated with transcatheter coil embolization regardless of type [3], endovascular treatment of wide-necked aneurysms is not yet well established and may require the use of more sophisticated techniques. These techniques, originally conceived in neurointerventional practice, are based on balloon- or stent-assisted embolization. During these procedures, a suitable device is released within the feeding vessel across the neck of the aneurysm to prevent coil prolapse during deployment and facilitate denser packing of the aneurysm, with the aim of reducing the likelihood of parent-vessel embolization. Stents employed are either self-expandable (bifurcation and segmental artery) or balloon-expandable (main stem) depending on the reference vessels and operator preference [7,11].

Complications associated with these techniques include artery dissection, thrombosis, and rupture; microthrombi embolization; and renal infarction [8,11]. Endovascular techniques are not often used in distal aneurysms, since the smaller the reference vessel, the harder it is to release a stent or use a balloon without damaging the vessel wall or inducing thrombosis.

The use of stent graft has also been reported for treatment of aneurysms beyond the first renal artery division but has a low success rate in terms of sparing the segmental artery and parenchyma [10,12,13].

## Case presentation

**Figure 1:**
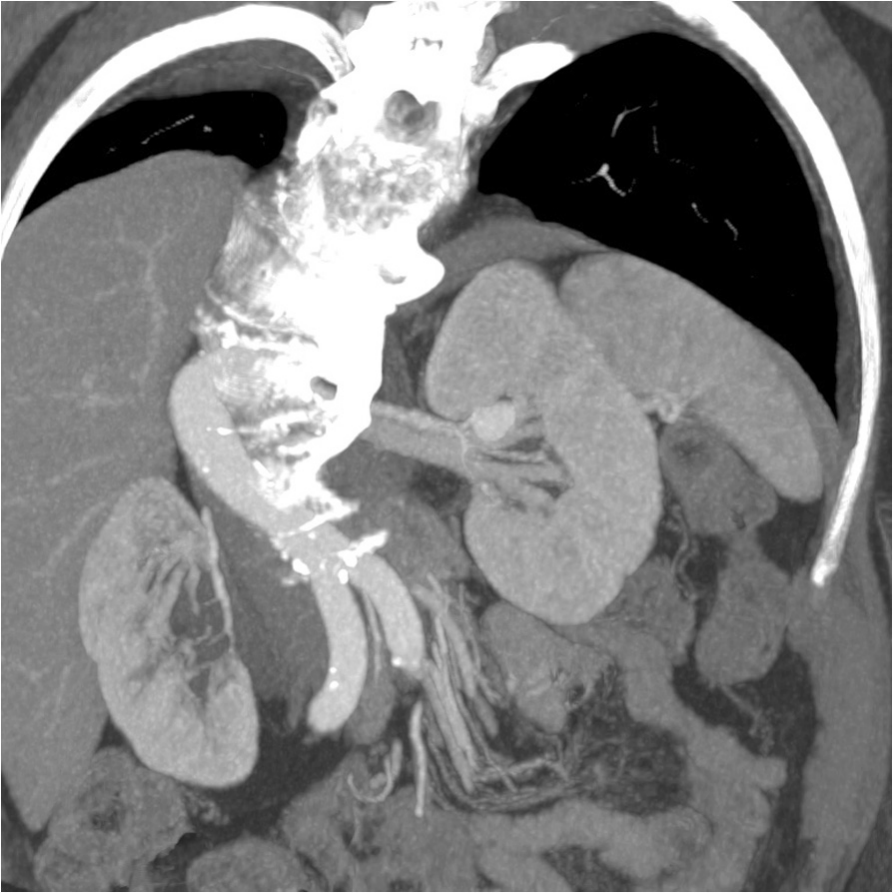
**Contrast-enhanced abdominal computed tomography.** Oblique coronal multiplanar reconstruction shows the aneurysm in the upper portion of the left kidney, originating from the anterior segmental artery.

**Figure 2:**
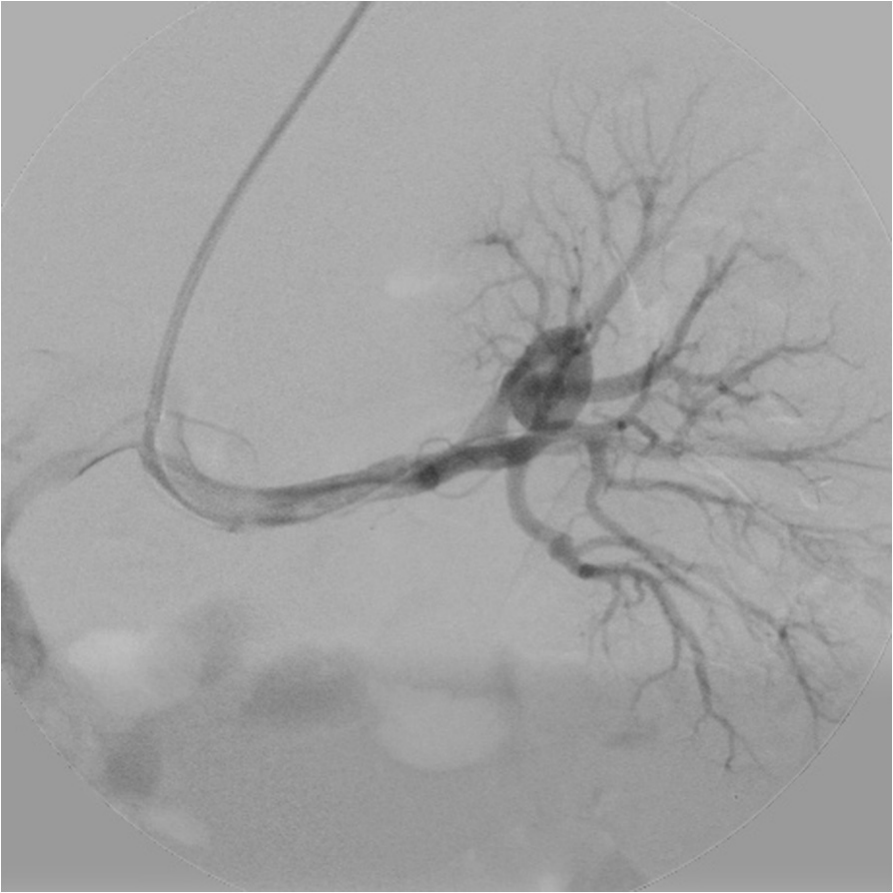
**Digital subtraction angiography.** Selective left renal injection through a transbrachial multipurpose 5-Fr catheter, with rapid injection of a wide-necked aneurysm originating from an upper-pole anterior segmental artery.

**Figure 3:**
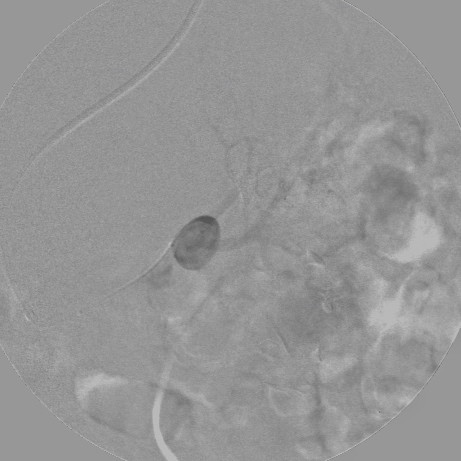
Superselective contrast injection into the aneurysm through a dedicated microcatheter coaxially advanced inside the larger 5-Fr angiographic mother catheter, immediately before the coil deployment.

A 74-year-old woman presented to our hospital emergency room with sharp left flank pain radiating posteriorly. She had experienced three similar episodes over the last 9 months. During one of the episodes, the patient had undergone clinical examination and abdominal ultrasound, which showed a renal intraparenchymal aneurysm about 10 mm in diameter.Her medical history was remarkable for severe chronic obstructive pulmonary disease, diabetes, and hypertension, all treated medically. After the patient was admitted, a color Doppler ultrasound (CDUS) and a multidetector computed tomography (MDCT) scan confirmed an 18-mm saccular aneurysm of the left renal anterior segmental artery. The aneurysm was characterized by a wide (12-mm) neck and the absence of intraluminal thrombus or parietal calcification (Figure 1).A multidisciplinary consultation that included general and vascular surgeons, urologists, and interventional radiologists was held, in which aneurysm morphology, the absence of calcifications, and the propensity of the aneurysm to enlarge were considered. In light of the patient’s performance status and comorbidities, the decision was made to treat the aneurysm with endovascular exclusion. After the patient provided informed consent, the embolization was performed under local anesthesia via transaxillary access. After selective catheterization of the left renal artery, digital subtraction angiography (DSA) was performed using a 5-Fr Cobra 1 angiographic catheter (TEMPO®AQUA®, Cordis Corp., Bridgewater, NJ, USA) coaxially introduced into a 6-Fr flexible, long-sheath introducer (Flexor® CheckFlo® Introducer; Cook Medical, Bloomington, IN, USA) to assess the relationship between the aneurysm and the left renal vasculature on multiple projections (Figure 2). After introducing a coaxial neurointerventional microcatheter (Penumbra PX400™ Delivery Microcatheter; Penumbra Inc., Alameda, CA, USA) into the aneurysmal sac, superselective DSA was performed to confirm the absence of branches originating from within the sac, and a slow, swirling flow inside the sac was observed (Figure 3).

To protect the renal vasculature, three complex-shaped bare nitinol microcoils (Penumbra Coil 400™ 18 mm × 57-cm and 15 mm × 57 cm Complex Standard and 13 mm × 48-cm Complex Soft; Penumbra, Inc.) were then sequentially deployed in the aneurysm until it was densely packed to 10.5% of the aneurysm volume. Each coil was detached only when angiography clearly showed complete and exclusive coiling inside the sac. The first coil, which was used to prevent coil migration through the neck into the parent artery, was the stiffest available and was sized slightly larger than the aneurysmal dome, thus creating a cage through which the other, more flexible coils could be safely deployed.

**Figure 4:**
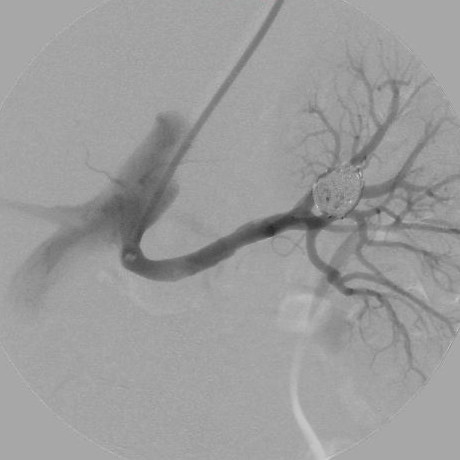
**Selective left renal artery angiography after coil deployment and detachment.** There is a compact appearance of the coils and complete filling of the aneurysm with downstream vascular-tree preservation.

**Figure 5:**
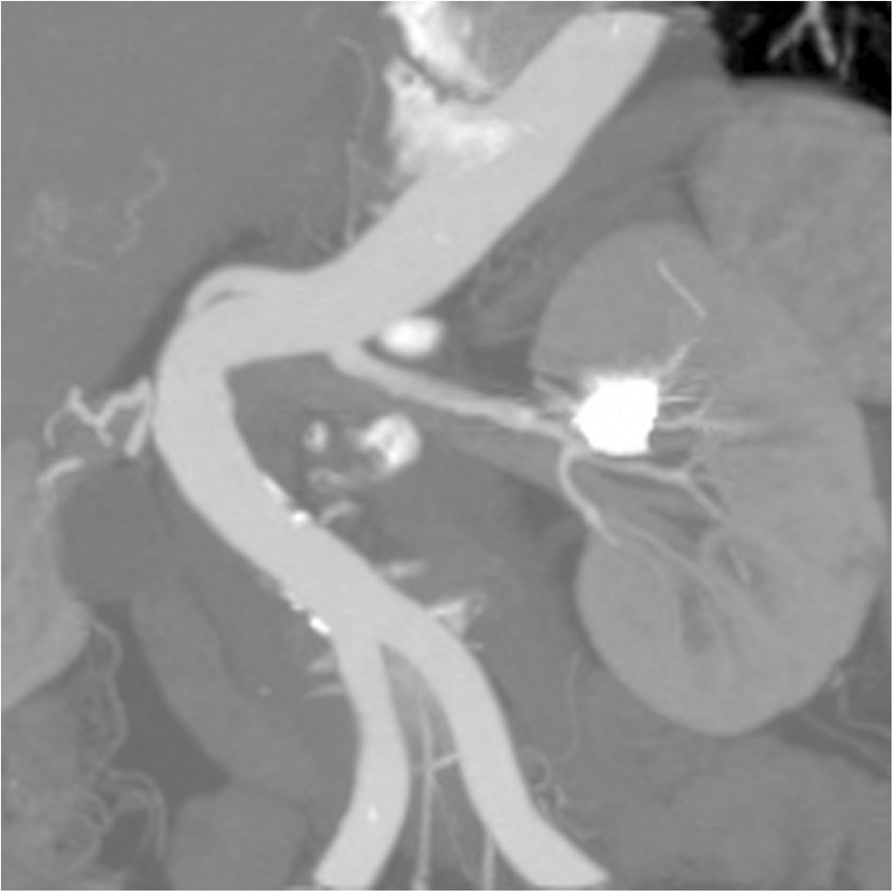
**Abdominal contrast-enhanced computed tomography 1 month after the procedure.** Oblique coronal multiplanar reconstruction clearly shows the aneurysm exclusion from complete coil filling and normal parenchymal contrast enhancement without evidence of segmental ischemic damage.

The final DSA demonstrated complete embolization and regular renal parenchymal opacification without parent-vessel embolization or thrombosis (Figure 4). The patient was asymptomatic at the time of discharge, 48 h after the procedure. Renal function, as evaluated by estimated glomerular filtration rate, was similar before (89 mL/min/1.73 m^2^) and after the intervention (92 mL/min/1.73 m^2^). The patient was followed up monthly with clinical examinations and laboratory tests. Her blood pressure remained unchanged, and her previous therapies were maintained. CDUS performed at 3 months and MDCT performed 1 and 3 years after the intervention confirmed preservation of the superior segmental artery and normal parenchymal perfusion (Figure 5).

## Conclusions

In the present case, considering the patient’s comorbidities and location of the aneurysm, surgical repair would have been very invasive and would likely have required bench surgery, so endovascular exclusion was preferable. The anatomical features and location of the aneurysm, and the need for accurate coil deployment before release, justified the use of this new type of neurointerventional detachable coil, which has not previously been reported to treat RAA. A high packing density, with fewer coils and shorter procedure time than are required for other procedures using different materials, was also obtained.

To our knowledge, this is the first case of a visceral aneurysm treated with the Penumbra Coil 400™ Embolization System (Penumbra Inc.) that includes 3 years of follow-up. These 0.020-inch coils are larger in diameter than other neurointerventional coils and are inherently softer because of their diameter (softness increases by the third power of the diameter) and because of the different thickness of the nitinol stretch-resistant wire. The coil-within-a-coil design increases the ability of the device to occupy the aneurysmal sac with less risk of misplacement [14].

As previously reported, in the treatment of complex renal aneurysms in difficult locations, a neurointerventional coil system may facilitate complete sac embolization with lower morbidity and less risk of decreased renal function than can be achieved with surgery or other endovascular techniques. The Penumbra system in particular, because of its conformability and greater length-volume ratio, allows rapid and complete embolization with less risk of parent-vessel damage.

More cases should be treated in this manner to validate the safety and efficacy of this system.

## Consent

Written informed consent was obtained from the patient for publication of this Case report and any accompanying images. A copy of the written consent is available for review by the Editor of this journal.

## Abbreviations

RAA: Renal artery aneurysm; MDCT: Multidetector computed tomography.

## Competing interest

The authors declare that they have no competing interests.

## Authors’ contributions

GMV and MR drafted the report, contributed to concept, and cared for the patient. GO and CDN drafted the report, and approved the final version of the manuscript. AR and FL contributed to concept and design and made relevant corrections. All authors read and approved the final manuscript.

## Pre-publication history

The pre-publication history for this paper can be accessed here:

http://www.biomedcentral.com/1471-2490/14/42/prepub
